# “*He Who Relies on His Brother's Property Dies Poor”*: The Complex Narratives of Livestock Care in Northern Tanzania

**DOI:** 10.3389/fvets.2021.749561

**Published:** 2021-11-03

**Authors:** Alicia Davis, Jennika Virhia, Joram Buza, John A. Crump, William A. de Glanville, Jo E. B. Halliday, Felix Lankester, Tauta Mappi, Kunda Mnzava, Emanuel S. Swai, Kate M. Thomas, Mamus Toima, Sarah Cleaveland, Blandina T. Mmbaga, Jo Sharp

**Affiliations:** ^1^Social and Political Sciences/Institute of Health and Wellbeing, University of Glasgow, Glasgow, United Kingdom; ^2^The Nelson Mandela African Institution of Science and Technology, Arusha, Tanzania; ^3^Centre for International Health, Otago Medical School, University of Otago, Dunedin, New Zealand; ^4^Institute of Biodiversity, Animal Health and Comparative Medicine, College of Medical Veterinary and Life Sciences, University of Glasgow, Glasgow, United Kingdom; ^5^Paul G. Allen School for Global Health, Pullman, WA, United States; ^6^Kilimanjaro Christian Medical Centre, Kilimanjaro Clinical Research Institute, Moshi, Tanzania; ^7^Kilimanjaro Christian Medical University College, Moshi, Tanzania; ^8^Ministry of Livestock and Fisheries, Dodoma, Tanzania; ^9^School of Geography and Sustainable Development, University of St. Andrews, St. Andrews, United Kingdom

**Keywords:** health seeking behaviours, One Health, livestock health, KAP, East Africa

## Abstract

**Background:** Endemic zoonoses have important impacts for livestock-dependent households in East Africa. In these communities, people's health and livelihoods are severely affected by livestock disease losses. Understanding how livestock keepers undertake remedial actions for livestock illness has the potential for widespread benefits such as improving health interventions. Yet, studies about livestock and human health behaviours in the global south tend to focus on individual health choices. In reality, health behaviours are complex, and not solely about individualised health experiences. Rather, they are mediated by a range of “upstream” factors (such as unequal provision of services), which are beyond the control of the individual.

**Methods:** This paper presents qualitative research conducted from 2014 to 2019 for a study focused on the Social, Economic, and Environmental Drivers of Zoonoses in Tanzania (SEEDZ). Qualitative data were collected via focus group discussions, community meetings, informal interviews, formal in-depth interviews, observations and surveys that addressed issues of health, disease, zoonotic disease risks, and routes for treatment across 21 villages. Thematic analysis was carried out on in-depth interviews and focus group discussions. Conceptual analyses and observations were made through application of social science theories of health.

**Findings:** Livestock keepers undertake a range of health seeking strategies loosely categorised around self and formal treatment. Two key themes emerged that are central to why people make the decisions they do: access to resources and trust in health care providers. These two issues affect individual sense of agency which impacts their ability to act to improve livestock health outcomes. We suggest that individual choice and agency in veterinary health seeking decisions are only beneficial if health systems can offer adequate care and health equity is addressed.

**Significance:** This study demonstrates the value of in-depth qualitative research which reveals the nuance and complexity of people's decisions around livestock health. Most importantly, it explains why “better” knowledge does not always translate into “better” practise. The paper suggests that acknowledging and addressing these aspects of veterinary health seeking will lead to more effective provision.

## Introduction and Background

Across Africa, over 70% of people rely on livestock for their livelihoods (1). Within East Africa, the reliance on livestock translates into multiple forms of livestock-based livelihoods such as pastoralism, agro-pastoralism, and small-scale farming (2). Sixty percent of rural households in Tanzania derive income from livestock which comprises 22% of total household income (3). Yet, livestock face numerous health challenges including, but not limited to endemic zoonoses such as brucellosis, Q-fever, Rift Valley fever, and anthrax. These diseases can threaten livestock-based livelihoods by directly affecting human and animal health (4, 5) and also indirectly through livestock production losses (6). Thus, there are linkages between human, animal, and environmental health, commonly referred to as One Health, which framed this research. As studies of One Health have shown, the health and well-being of one's livestock have broader socio-cultural impacts connected to human health and well-being as well (7, 8).

The issue is further compounded by limited access to formal human and livestock health care in remote rural communities (9) as well as by other livelihood and infrastructural constraints (10–12). These factors cause disproportionate economic and social burdens on the rural poor, leaving them and their livestock more vulnerable to disease (4, 13, 14). Livestock keepers in Arusha and Manyara Regions of northern Tanzania often have to make difficult decisions within a veterinary health system which imposes limitations on the treatment options available to livestock keepers. As presented in detail below, both health systems in Tanzania are shaped by health policies that stipulate public-private partnerships, with overstretched state services, and a lack of private service to fill the gaps (15). This is reflective of similar health constraints faced by the rural poor across the globe (16). Thus, understanding the impeding factors and pathways taken by livestock keepers for livestock care (including for ill health caused by zoonoses) is key to safeguarding human health, in addition to designing effective policy and disease management support.

Attempts to understand health behaviours often draw on measuring levels of knowledge, awareness, practises and beliefs in relation to a particular health issue. “Health seeking behaviour” (HSB) studies for example are used to describe why, when and how individuals, social groups and communities seek access to health care services (17, 18). They achieve this by following the sequence of remedial actions undertaken for illness, from the recognition of symptoms through different types of help seeking until they feel healed or capable of living with their condition (17). Studies on health seeking behaviours overwhelmingly relate to human health, and most conceptual frameworks seeking to explain health behaviour and access to care directly relate to human experiences and their choices [see for example Muela et al. (19) and Obrist et al. (17)]. Studies on animal health seeking behaviours similarly focus on individual human decisions and actions taken to manage animal ill health [see Awosanya and Akande (20); G/hiwot et al. (21)]. Understanding how people seek healthcare for their livestock has important implications for human health (e.g., in the case of zoonoses), for human livelihoods, as well as in demonstrating the intricate social and cultural connectivity between animals and humans.

The comon issue in the application of both animal and human health seeking is the tendency to focus attention on the individual decision-maker (22) with less consideration of the systemic constraints which may impact their health decisions. For instance, many interdisciplinary studies of health in the global south rely on knowledge, attitude, and practise (KAP) surveys [sometimes referred to as knowledge, attitude, behaviour, and practise (KABP) surveys] (23, 24). KAP studies are commonly utilised in interdisciplinary approaches to understand complex systems, and often aim to collect quick “qualitative context” through interviews or focus group discussions [see Caudell et al. (9)]. However, while offering important insights into a particular health issue, this often happens at the expense of long term, in-depth understanding about wider social and cultural factors that both constrain or enable individual action. This point has been most powerfully made in Farmer's (25) influential work on Tuberculosis and HIV/AIDS:

The countless Knowledge, Attitudes, and Practises surveys and AIDS educational interventions derived from them have not achieved their aim, and to say so *is not to object to* AIDS *education* […] But show us the data to suggest that, in settings where social conditions determine risk for HIV infection, cognitive exercises can fundamentally alter risk. We know that risk of acquiring HIV does not depend on knowledge of how the virus is transmitted, but rather on the freedom to make decisions. Poverty is the great limiting factor of freedom. [(25), p. 40, emphasis added]

As Farmer suggests, the ability to make “appropriate” decisions around health does not solely depend on knowledge, but also on individual ability (or agency) to make choices within enabling or constraining contexts in which people live. Poverty is the greatest limiting factor for agency, but it is far from the only one. Studies that are predicated on identifying discrete variables (or individual actions) that can be pinpointed for “risk reduction,” “awareness raising,” or “knowledge building” (26) can result in a “straightjacket” that leads to a “narrowing of the social world” [(14), p. 14] thus missing the hetereogeneity in which health, and social life more broadly, occurs [see also Bardosh (27), for mixed methods approaches to study about NTDs that expand beyond KAP]. Without an understanding of the sociopolitical contexts within which individuals make decisions (28), and particularly health decisions, and indeed veterinary health decisions, there is a danger that HSB studies, and KAP studies as an example of these, overemphasise the *agency* of individuals to act as capable and rational actors while ignoring the ways contextual issues and systemic barriers influence individual health, health related behaviours, and broader access to care (25). Individual actions, “rational” economic behaviours and decisions thus become the focus for health interventions and health actions (25, 29) which can subsequently lead to a belief that people are behaving “irrationally” when they do not follow “expected” behavioural norms. This is true for both human and veterinary health. As Parker et al. (30) contend that key insights about how people experience health and illness are only gained through longer periods of time and investment in ethnographic engagements which in turn affects broader debates about and investments in health.

Drawing on in-depth qualitative data from Tanzania about health, health seeking behaviours, and care for livestock, this paper seeks to go beyond a traditional KAP study to reveal how “everyday” experiences of livestock health are structured not only by individual behaviours and preferences but also by key structural factors, including systemic health inequities^1^ and challenges within veterinary health systems. Understanding these wider, contextual factors can reveal the reasons why better knowledge or attitudes towards risk may not lead to changed practise because the individual is not able to change the conditions that constrain their actions (as the HIV/AIDS example above provides). The paper will contextualise the strategies adopted by livestock keepers to manage the health of their livestock thus providing a deeper understanding of factors influencing veterinary based health seeking behaviours.

### Tanzanian Health Landscape (Human and Veterinary)

**Figure 1:**
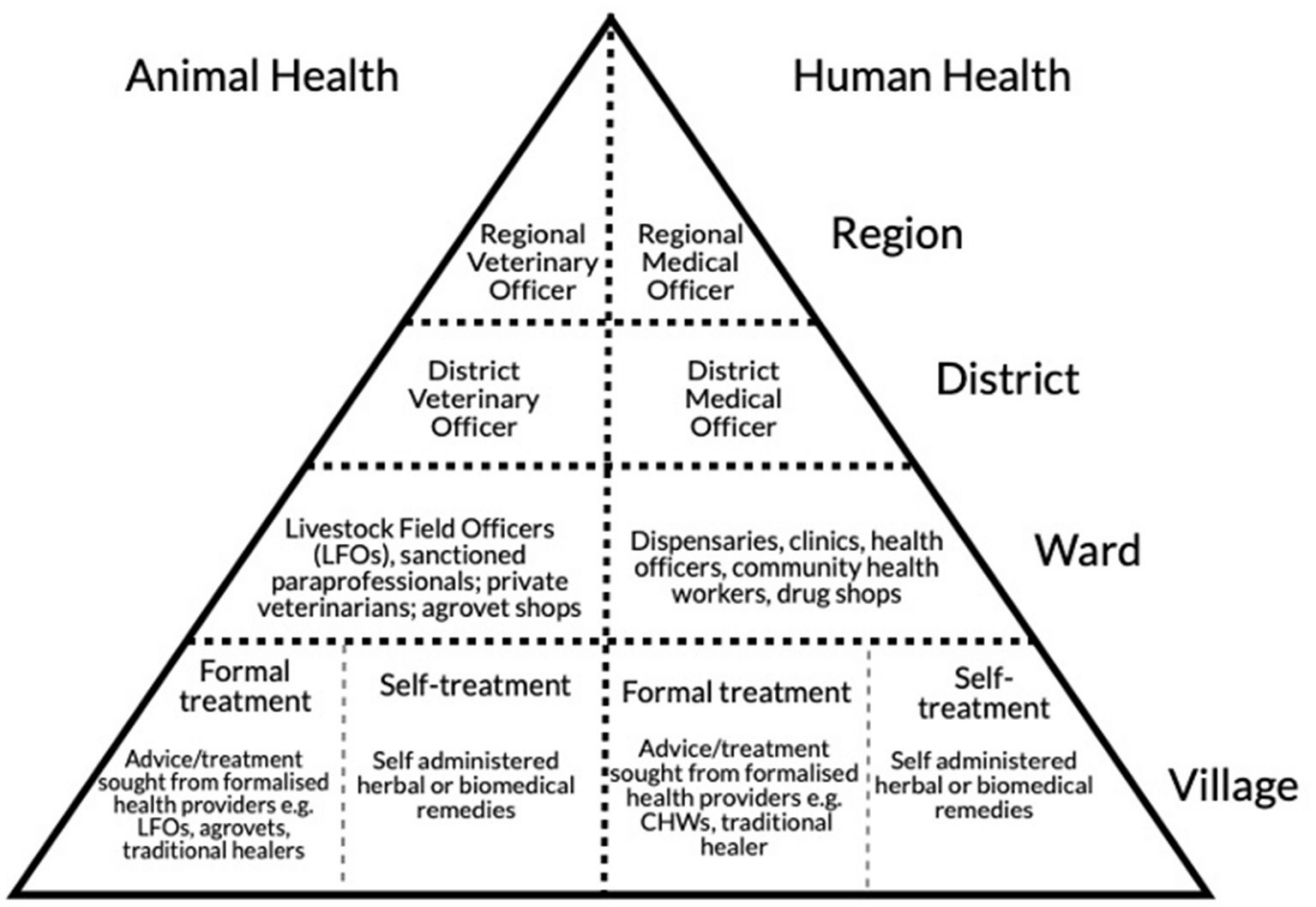
Tanzanian veterinary and human health system structure. The country is divided into distinct administrative units, with the Region being the largest. Each Region is comprised of up to 7 Districts. District health administration includes a formal District Veterinary Officer (DVO), and District Medical Officer (DMO) who are trained degree holding professionals and who lead a team of district [(para)veterinary and medical] officers. Wards are administrative units that encompass 2–6 villages, with each ward or village acting as the central location for extension services: for example veterinary, agricultural, and medical. Ward officers for veterinary health include Livestock Field Officers (LFOs) who are trained (at certificate level or higher) in livestock health, livestock production, range management and who serve multiple villages. Ward officers for human health include clinical officers (who work in dispensaries and throughout the health system), technicians, and community health workers.

The structures of both the human and veterinary health system in Tanzania, established at independence in 1964 under the “African socialist” reforms of the Nyerere presidency (1964–1985) form a strong edifice from which care is organised in the country. This underlying structure was based on centralised government authority with district intermediaries who supervised field extension services in rural communities, budgeted by national health and veterinary ministries (see Figure 1). While the basic frameworks (and underlying bureaucracies of management) were established during the post-independence socialist period, the ensuing Structural Adjustment era in the 1980s (spearheaded by the International Monetary Fund) led to substantial changes in the delivery of livestock and human health in Tanzania. This primarily included: decentralisation of government authorities, defunding of public services, and the increasing privatisation of health provision (yet with limited capacity to increase private services) (32, 33). The veterinary health system parallels the human health system but has been subject to even greater privatisation. This is evidenced through the emphasis of public-private partnerships for meeting veterinary health needs in the most recent livestock policies (34). With government services particularly underfunded (33) and the private sector lacking in service providers, the veterinary health system has left rural areas largely underserved (35). In these areas, the number of livestock greatly outstrips the capacity of the health providers available (33). For example, within Ngorongoro District, an area with high livestock density, 73% of pastoralists reported having no access to extension services (33) (which includes basic animal health services). Thus, the current system often falls short of meeting local human or veterinary health needs, with public provision of veterinary care in particular facing striking disadvantages for meeting broad scale animal health needs (36).

As a result, there are numerous challenges in providing adequate health services when livestock are ill, or in providing sufficient health information for prevention measures. There are also significant challenges for livestock keepers in accessing services when they are available. This is especially salient for rural livestock keepers who live in complex environments with increasing pressures from large scale land use change and climate change or conversion of grazing lands to farms or conservation areas (11, 37–41). This is further compounded by conflicting expectations of both government and citizens about who has the key responsibilities for service provision, including for vaccination against endemic and epidemic diseases [see United Republic of Tanzania (URT) Livestock Policy (34)]. There is also a long and complex history of neglect from and mistrust of available veterinary systems and experts spanning back first to colonial regimes, through the post-independence, socialist based system, and lasting into present day (8).

Within the structure of the veterinary system, uneven access to government services (for which most rural livestock keeping communities rely upon) exists depending on the type of livestock keeping system. For example, it is common practise in Tanzania for there to be one livestock field officer (LFO) per ward regardless of the number of livestock living in that ward^2^. Thus, smallholders, who keep far fewer animals than pastoralists, and in geographically smaller villages often have easier access to service providers and veterinary supplies due to closer proximity. Rural infrastructure (including roads, cellular service, water supplies, schools) tends to be poorer in districts where pastoralists reside (42). In the absence of access to government *or* private livestock health services, livestock keepers are often left with no choice but to manage livestock disease completely on their own, sometimes with little to no advice much less direct hands-on evaluation available and with varying conceptions of self “expertise” (43).

In order to understand how livestock keepers manage animal ill health in light of varying provision of and access to services, it is important to map the range of options that livestock keepers have within the Tanzanian veterinary health system. The very real set of constraints and challenges within the system affects how people make decisions about the care for their animals. What emerges is a range of veterinary care and health seeking behaviours (HSB) that highlight the ways in which livestock keepers mediate that veterinary care. These HSB span from self-reliance for treatment, i.e., “self-treatment” to utilisation of diagnostic based care offered by trained private or public veterinary practitioners. Both formal and informal health care options exist within this suite of HSB. In practise, these are often utilised simultaneously as there are not simple choices of “formal” vs. “informal” nor “self-treatment” vs. supervised care. Furthermore, these options are not mutually exclusive or exclusionary. For example, and as our data will show below, in the case of agrovets a livestock keeper may purchase drugs based on personal prior experience and ethnoveterinary knowledge *or* may ask the seller for advice and guidance. Similarly, LFOs may be consulted for advice via phone or be called out to examine a sick animal (but, as our data will show, usually as a last resort). Thus, while we present the typical binary framework of care, we also point to the messy reality and strategic practise that often occurs in daily life (Table 1). An outline of animal health providers and their roles is provided in Table 2.

**Table 1:** Typical types of treatment options are often categorised as “self-treatment” or reliance on more “formal” treatment channels (biomedical here refers to treatment options based in the formal (western) scientific tradition, whereas local refers to informal, local, traditional, or management based ethnoveterinary treatments).

**Types of treatment options**			
**Self-treatment**		**Formal**	
Biomedical	Agrovet shop (drugs bought based on experience)	Biomedical	Agrovet shop (advice sought from formally trained seller)
	Market drug sellers		Evaluation/assessment from LFO or DVO
	Advice (from social network or animal health providers)		Evaluation/assessment from private vet or paraprofessional (including informal providers, such as CAWHs)
	Self (based on past experience)		Regional vet testing facilities
Local practises	Use of local herbs or remedie	Local practises	Local herbalists, healers
	Behavioural/management strategies		Local experts in birthing

*Importantly, we include herbal and traditional healers as “formal” options as, although they are not government sanctioned or trained with biomedical credentials, they are widely recognised among livestock keepers as formalised providers of treatment and advice [see Langwick (44) for further discussion on the regional importance and legitimacy of traditional healers for therapeutic interventions]. Health seeking pathways often begin with self-treatment and may end up with individuals seeking formal treatment if the problem persists or escalates to an unmanageable level. Simultaneous use of treatment options also commonly occurs*.

**Table 2:** Categorisation of formal and informal animal health service providers in Tanzania [adapted from Virhia (45)].

**“Expert”**	**Definition**
Veterinarians (public and/or private)	Individuals who hold a degree in veterinary medicine or its equivalent from a veterinary institution recognised by the veterinary statutory body (The Veterinary Council of Tanzania) (46)
Veterinary Paraprofessional (VPP)	Individuals who have received formal training at diploma level in animal health level from training institutions accredited by the appropriate government agency or the veterinary statutory body and the activities that they are permitted to conduct will reflect their level of formal training (47)
Veterinary Paraprofessional Assistant (VPPA)	Individuals who have received training at certificate level in animal health from training institutions accredited by the appropriate government agency or the veterinary statutory body and the activities that they are permitted to conduct will reflect their level of formal training (47)
Community Animal Health Workers (CAWH)	CAHWs can be considered as distinct from VPPs/VPPAs as they generally do not have a certificate from a government accredited training institution. They are mainly livestock keepers who are nominated by the community and trained (by government officials, NGOs or farmer organisations) in basic animal health techniques (such as vaccination and deworming for instance) and who deliver a limited range of veterinary services to their communities.
Livestock Field Officers (LFO)	Individuals appointed by the government to provide livestock extension and advisory services at the village or ward level. LFOs should receive formal training at either the diploma or certificate level in animal production and range management from training institutions accredited by the appropriate government agency.
Local experts	Those without any government recognised qualifications but are known by others in their community as having knowledge through experience.
Agrovets	A supply store for farmers selling veterinary products (including medications, animal feed, supplements pesticides, vaccinations) and agricultural products (including seed, fertilisers and herbicides). Individuals working in agrovets are often viewed as a source of knowledge and advice on livestock and agricultural issues. Agrovets may sometimes be owned and run by LFOs.
Traditional healers	An umbrella term used to describe healers who call upon divination and spirituality among other remedies to solve disequilibrium among afflicted individuals (48).
Situational experts	Those who have knowledge about particular animal health issues such as birthing, or specific diseases.

## Methods

This paper presents data from the “Social, Environmental and Economic Drivers of Zoonotic disease” (SEEDZ) project conducted in northern Tanzania from 2015 to 2019. SEEDZ data collection included a large cross-sectional study of human and livestock zoonotic disease risk in 21 villages across ten districts in two regions of northern Tanzania (Arusha and Manyara) in an area of 66,461 km^2^ and within semi-arid and sub-tropical agro-ecological zones (49). The two regions have a population of 3.1 million people and ~16% of all cattle and 26% of all sheep and goats in Tanzania (50, 51). Social science data collection was built into the cross-sectional design and included mixed qualitative and quantitative tools applied at community and household levels. A detailed overview of the cross-sectional study design and methods can be found in Ahmed et al. (6) and de Glanville et al. (2).

### Site Selection

Villages were stratified based on primary livelihood activity and included pastoralist communities, dominated by transhumant livestock production, and “mixed” communities that practise both livestock production and crop farming [see de Glanville et al. (2)].

**Figure 2:**
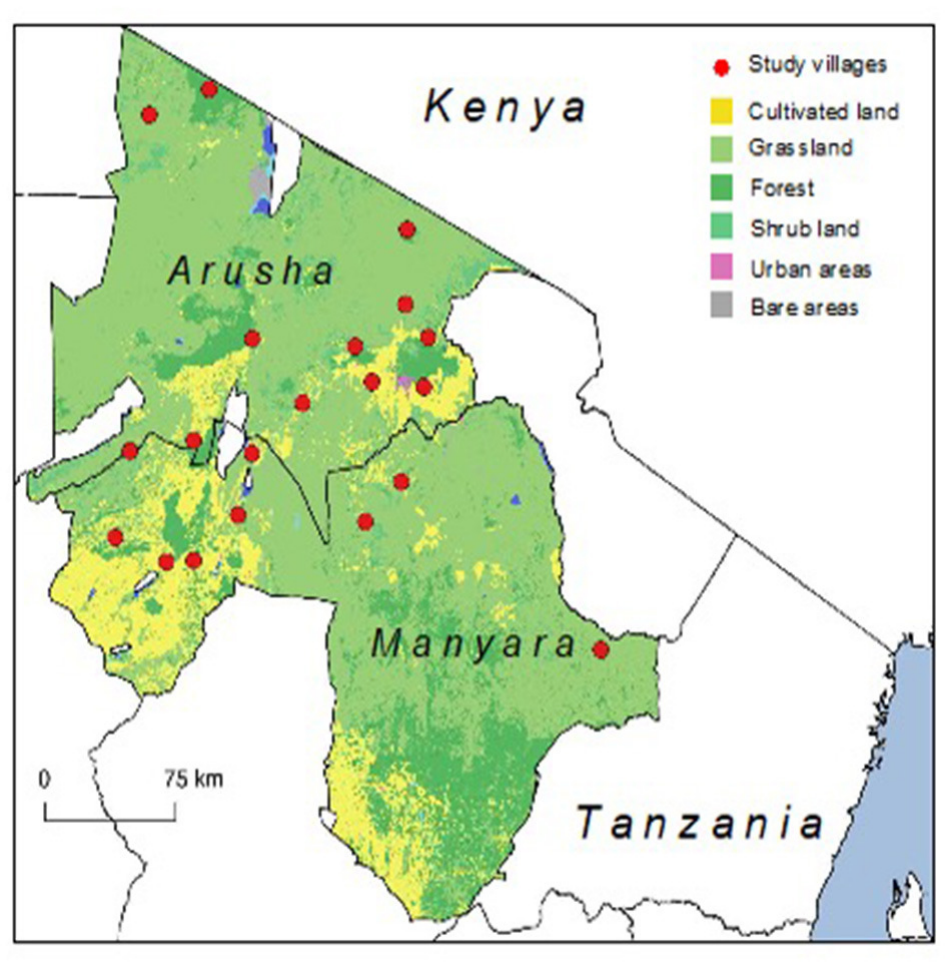
Map of study regions and villages in northern Tanzania. Land classifications denoted in Arusha and Manyara Regions [from de Glanville et al. (2)].

The categorisation of villages was carried out with district administrators [e.g., District Veterinary Officers (DVOs)] and 11 pastoralist and nine “mixed” villages were selected, with one periurban “mixed” village on the outskirts of Arusha city selected for piloting the study. All villages were included in data analysis as methods were not modified after piloting. Areas for household sampling were determined in agreement with village authorities by random selection of 2–3 sub-villages (administrative units that divide villages, with an average village having 3–5 sub-villages). See Figure 2 for a map of the study regions and villages.

### Quantitative and Qualitative Data Collection

To maintain our long-term relationships with communities, our data collection built on previous studies conducted by the researchers on the causes of fever in the region and included several overlapping villages. Further, we contribute detailed ethnographic experience in the study area based on the authors' individual research in the country on a host of health and non-health related issues (totalling over 20 years). Thus, data collection tools and qualitative data particularly, is couched in long-term ethnographic study in the country. Household questionnaires were broad surveys that included information on household demographics, economics, livestock management, and livestock health. They also included questions about household decision-making, gender roles around livestock management, and zoonotic disease awareness. Qualitative data collection was carried out in each sub-village using focus group discussions (FGDs) (average of 10–15 participants each) and in-depth, semi-structured interviews (IDI) (see Table 3) wherein participants and researchers were provided space to answer freely, open ended questions with on-the-fly follow-up questions asked as the conversation dictated. Key members of each community were identified via village officials (such as village chairpersons and executive officers) and were invited to participate. They included: village leaders (governmental, traditional and women's leaders), and widely respected members of the community. IDIs also included local health (veterinary and human) providers. FGDs were overwhelmingly gender segregated (with only seven mixed groups out of 57 FGDs) in order to provide women space to speak freely amongst their peers, a common practise in patriarchally dominated communities. Follow up interviews were conducted in a selection of eight villages between July and October 2018 to further explore health seeking behaviours for livestock and human illness. These were selected opportunistically from previous surveys or in-depth interview participants and based on field team capacity and budget and the respondent's time availability and willingness to talk to us again. Interviews were audio recorded (when consented to by participants), transcribed and translated from Swahili or local language (primarily Maasai and Iraqw languages) into English by project research assistants. Any discrepancies in translation were minimised through continued discussion with translators and alignment of vocabulary and commonly used terms. Translators were often the same field team members conducting the interviews or participating in broader data collection, thus had a familiarity with interview questions, cultural and language contexts, and commonly used terminology. We also made repeat visits for follow up interviews to a selection of interviewees to build trust in communities, verify data and for data triangulation. Where interviews were not audio recorded, in-depth handwritten notes were taken by a dedicated note taker and typed for translation and analysis. All materials were stored as password protected files and secured as per University of Glasgow and the National Institute of Medical Research in Tanzania ethical approvals (see below for details). Personal or identifying information such as names were removed from all transcripts. All identifiers, including village names, were removed for presentation in the findings below.

**Table 3:** Qualitative interviews conducted across study sites.

**Interview type**	**No. villages**	**Pastoral**	**Agro-pastoral**	**No. interviews**	**Total participants**
Focus group discussion (FGD)	21	12	9	57	575*
In-depth interview (IDI)	21	12	9	35	35
Follow on (FO)	8	6	2	58	58

*numbers are an estimation as there was often a flow of people in and out of interviews given they were often in outdoor public meeting areas with people leaving early or joining late. Average interview size was 10 participants. Verbal consent was given for any participant joining.

### Analysis

Qualitative analysis was conducted using NVivo™ (version 12) ethnographic software (QSR International) and by combining inductive and deductive thematic analyses (52, 53). We created a coding framework based on interview questions which focused on actions taken in response to livestock ill health and livestock disease risks. After an initial reading of the interviews, iterative codes were then added as emergent themes arose. Coding was conducted by three of the authors (AD, JV, and JS) with regular cross checking, double coding, and discussion for consistency, concurrence and agreement. Key themes included summary descriptors of participants health seeking behaviour, which we categorised into “self” treatment or “formal” treatment. A quantified summary of key themes relevant to this paper was deduced after several rounds of thematic coding (see Table 4). We further categorised emergent HSB into themes that described underlying patterns of sentiment, behaviour, and experience. Further analysis of descriptive themes, when examined with broader socio-political contexts, revealed underlying motivations or influences to HSB and include aspects of agency, access, and trust, and are presented in the discussion.

**Table 4:** HSB decision narratives demonstrating (1) the causal factors leading to specific health decisions, (2) the subsequent health seeking actions (and their variants) and (3) the key contextual factors which influence health decisions.

**Health seeking behaviours: possible decision narratives**		
**Causal factor leading to decision**	**Actions and variants**	**Contextual influences**
**Self-treatment HSB**		
Sick livestock	•Self-diagnosis based on observation of livestock behaviour and clinical signs •Identification of known diseases	•Indigenous livestock breed •Familiarity of disease •Funds available
Biomedical preference	•Use of drugs known to be effective through purchase or stocks kept at home •“Trial and error” use of drugs kept at home •Agrovet: buying medication and self-administering to livestock •Calling other expert or social network for advice on diagnosis or drug use	•Advice from agrovets, livestock officers and social network •Past experience with positive outcome of specific drugs •Funds available
Local healing preference	•Collecting herbs, used for known diseases/symptoms •Herd management	•Local remedies known and used, but scepticism over effectiveness •Familiarity with disease/ailment •Familiarity with effectiveness of treatment
**Formal treatment HSB**		
Sick livestock	•Drawing on formal sources of advice from trusted expert	•Condition persists or worsens (*after* self-treatment)
Biomedical preference	•Calling the LFO •Calling a private vet or paraprofessional when selftreatment options exhausted •Agrovet: asking trained veterinary agrovet for advice on diagnosis/treatment options •Calling “non officially recognised” paraprofessionals such as CAHWs	•Exotic breed •For specific conditions (anthrax, black quarter) •For unfamiliar symptoms/ disease •During disease outbreak / vaccination
Local healing preference	•Calling in traditional healer or herbal expert	•Belief in traditional practises •Cost

*Causal factors initiate the need to seek remedial actions (i.e., a sick animal) and personal preference dictates whether biomedical or lay treatments will be chosen in the first instance. Choice is also heavily determined by contextual influences, such as prior experiences, familiarity, availability of providers, beliefs and breed of livestock which further highlight the complexity of factors that lead to certain health decisions*.

### Role of the Funding Source

The Biotechnology and Biological Sciences Research Council, Department for International Development, the Economic & Social Research Council, the Medical Research Council, the Natural Environment Research Council and the Defence Science and Technology Laboratory funded this research under the “Zoonoses and Emerging Livestock Systems” (ZELS) programme (BB/L018926/1 and BB/L018845/1). The funders had no involvement in the study design, data collection, analysis, or interpretation of the findings. The funders played no role in writing or submitting this paper.

### Ethics Statement

All participants provided written informed consent. The protocols, questionnaire tools and consent and assent procedures were approved by the ethics review committees of the Kilimanjaro Christian Medical Centre (KCMC/832) and National Institute of Medical Research (NIMR/2028) in Tanzania, and in the UK by the ethics review committee of the College of Medical, Veterinary and Life Sciences at the University of Glasgow (39a/15). Approval for study activities for each researcher was also provided by the Tanzanian Commission for Science and Technology (COSTECH) and by the Tanzanian Ministry of Livestock and Fisheries, as well as by regional, district, ward and villagelevel authorities in the study area.

## Findings

We present findings from a combination of data sources including FGDs, IDIs, surveys, and observations and field notes. Because of the overlapping and complex nature of the types of health seeking, we attempt to draw out subtleties through a summary of emergent themes, direct quotation from participants and ethnographic context. The first portion of the findings focuses on overall themes and trends, followed by discussion of the nuances of self-treatment (including intrinsic and extrinsic factors), and concludes with the experiences and contingencies of formalised care seeking.

### Mapping the Conditions and Contradictions of Health Seeking

To summarise overall findings about health seeking behaviours for livestock illness in our study communities, we first categorised participant's HSB as either “self-treatment” or “formal” treatment. We mapped known categories (as described in Table 1) against findings which emerged in the data (Tables 4, 5). Intrinsic factors for self-treatment options rely on one's sense of agency to treat animals themselves and include use of prior knowledge and experience, trial and error, utilising prior advice and preference for lay treatments. Extrinsic reasons include lack of access to formal services, prohibitive costs and mistrust in formal providers. In most cases, when self-treatment options are exhausted livestock keepers move onto formal treatments including seeking advice from agrovets and/or calling formal providers to come and evaluate and examine the animal as a last resort. While we initially categorised HSBs as either “formal” or “self-treatment” and “biomedical” or “local” treatment, in practise they are often in-between the two and commonly a combination of multiple options. For example, there is overlap between self-treatment and formal treatment, especially when agrovets are the primary source of advice for self-administration of treatment, as discussed above and in detail below. Moreover, we demonstrate the importance of *context* for informing health seeking practises. As the quotes below show, decisions are based on a range of interconnecting factors such as the availability of appropriate medication (the first quote) or of expert advice (the second) which we discuss in more detail throughout this section.

**Table 5:** Frequency table of themes in interviews.

**Frequency table of health seeking behaviours**				
**Treatment type**	**Treatment preference**	**Specific action mentioned**	**No. of FGDs theme emerged within (*****n*** **=** **41)**	**% of FGDs**
Self-treatment	Biomedical	**Drug purchase related:**
		General self-treatment	40	98
		Buying medicines from agrovet	18	44
		Buying medicines from a market	6	15
		**Information related:**
		Reliance on one's own past experience/self knowledge	32	78
		Self-treatment through a process of trial and error	21	51
		Seeking advice from one's social network	16	39
		Using preventative treatments such as dipping	17	41
	Local practises	Collecting and administering herbs/lay treatments oneself	19	46
		Using traditional herd management techniques for prevention or treatment	4	10
Formal treatment	Biomedical	Formal biomedical treatment including vaccinations or severe outbreaks	33	81
		Formal biomedical treatment excluding vaccinations or severe outbreaks*, i.e., everyday illness	18	45
		Treatment from LFO	10	24
		Treatment advice from agrovet	12	29
		Treatment *via* paraprofessional or community animal health worker (CAHWs)	7	17
	Local practises	Herbal/lay remedy expert	4	10
		Situational experts e.g., birthing	3	7

We only included interviews that had audio recordings and English transcripts for this component of the analysis, or a total of 41 of 64 FGDs. More than one type of treatment was typical for every interview analysed and no interview mentioned <4 types of specific actions. There were 6 interviews that described only self-treatment without formal treatment, whereas there were no interviews that mentioned formal treatment without self-treatment. ^*^Severe outbreaks refer to major disease outbreaks that span to community or broader than community wide incidence, and which often require government intervention and often lead to suspension of market activities, for example anthrax or RVF. We note that comparison should be made between formal treatment exclusive of vaccinations, to denote everyday use of formal systems.

**Q1**. *RESPONDENT (R)*^3^*: It is like this, there are lots of things that we do: first when the livestock is sick we normally bring medicine home, so when it gets sick you inject it [...] and if you don't have medicine inside [your home] you grind sisal and give it to the sick livestock. Later on you go to look for medicine in the shop*.

–Men's FGD participant, Village 18, agro-pastoral

**Q2**. *Interviewer (I): Because the LFO does not reach this village where do you get advice on issues related to livestock management? R: It is only up to the owner because if the LFO does not come, do you wait? No, you treat your livestock (by buying medicines) the way you see it is best. There is nowhere else to seek advice*. – Men's FGD participant, Village 6, pastoral

To illustrate the importance of context we describe respondents' health seeking patterns as decision narratives (Table 4). This incorporates: (1) the *causal* factors leading to specific health decisions, (2) the subsequent health seeking actions (and their variants) and (3) the key contextual factors which influence health decisions. The context in which livestock (and human) illness occurs is key to shaping an individual's sense of agency and thus informs subsequent health seeking behaviours. To highlight the interlinked nature of health seeking - livestock keepers often seek advice from those selling drugs in agrovets, and those advisors may be formally trained experts, (para)veterinarians or community animal health workers (CAHWs) or lay shopkeepers who only sell drugs as a business with no formal veterinary credentials. Thus, HSB in one category does not preclude the other and, most commonly, HSB narratives are overlapping, multi-stepped and multi-faceted.

Table 5 presents the frequency of specific treatment actions undertaken as part of the narratives of HSB discussed in our focus group discussions (*n* = 41). Nearly all participants described using self-treatment options (98%) when asked to describe what actions they normally take in response to livestock ill health. Specific questions included some variation of: “when you see signs of previously mentioned diseases, what is the first action you take?” These self-treatment options included 43% buying their own drugs from agrovets. Follow up questions investigated processes and further steps or actions as well as why these actions were undertaken. As part of this line of discussion, questions about where information or skills were gained in their assessment were often asked. Thus, these actions were often undertaken in conjunction with 78% of respondents indicating that they gained expertise through past experience and generational links (elder-youth transmission of ethnoveterinary knowledge) in treating their own livestock. Additionally, while self-treatment involves independent diagnosis or assessment of an animal's condition and then administration of medication to sick livestock, it does not preclude drawing on advice, either from social networks or from formal veterinary health providers.

In terms of formal treatment, 81% of participants reported using formal biomedical treatment options at some point during any number of livestock illness situations they freely named. Use of formal providers was also highly conditional, that is, once “vaccination events” or “disease outbreaks” were removed from health seeking scenarios, only 45% of participants in FGDs reported accessing formal biomedical treatments for when their livestock gets sick, and this was usually after all self-treatment options were explored. People with exotic breeds commonly stated their need to get veterinary attention straight away, though our data did not disaggregate HSB based on breed.

### Self-Treatment as a Complex HSB: Experience Narratives

Self-treatment encompassed an array of actions as explained in Table 4, ranging from intrinsic factors such as relying on own knowledge and past experience, trialling different drugs kept at home, lay practises, and seeking advice from friends, family and formal providers to extrinsic factors where ack of access to, and trust in, formal providers influenced health decisions. Other actions mentioned by a small number of participants include traditional herd management practises (such as isolating animals or preventing them from mixing with other herds) to prevent spread of diseases and buying medication from informal market sellers. These actions do not occur in isolation but rather are interwoven and repeated throughout the health seeking process, until the livestock gets better or dies. The words and experiences of the livestock keepers themselves (through our qualitative data) help create fuller HSB narratives and demonstrate the nuances and complexity of HSB practises. These are presented below.

#### The Self as Expert - Reliance on One's Own Knowledge and Past Experience

A sense of oneself as an expert, derived through experience in ethnoveterinary knowledge passed down through generations, provides a basis for some livestock keeper's belief in themselves as the most capable agents for providing care and treatment to their livestock. This was more commonly expressed in pastoralist study communities. However, when respondents were asked why they prefer to treat livestock themselves, many (across sites) referred to their extensive experience and knowledge in treating livestock:

**Q3**. *R1: I was not advised by anyone, I grew up in a livestock area, mostly [with] cattle so I learned from my father […] you know when you stay with the elders and do livestock activities together you get enough education*.

*R2: Absolutely, it is truly a school and enough experience. –* FO participant 28, agro-pastoral

***Q4***. *INTERVIEWER: So, you treat the livestock yourself? Rs: Yes*.

*INTERVIEWER: So, someone came and gave you a course that treatment is done in this way? Rs: no. R1: We gave ourselves the course. INTERVIEWER: So, you have learned from each other? Rs: Yes. –Men's FGD, Village 17, agropastoral*

People explained their own observational practises and skills in tracking their animals' conditions, behaviours, and health through their daily interactions with their livestock. Where self-assessment is possible and treatment options are known, people will follow this course.

**Q5**. *R1: What a woman can do is to tell if she has observed a certain sign in a cow, for example, if the cow has given little milk or there is some abnormality in the cow*. –Mixed FGD, Village 12, pastoral

A sense of self as an expert can lead to a variety of different health-seeking actions including trial and error with different drug treatments. Participants often had prior experience around “known” issues (such as East Coast fever) or in treating less well-known conditions, thus self-treatment often involves a process of experimentation using various drugs kept at home, or purchasing an array of drugs until one works.

**Q6**. *R3: We treat by guessing. If you think its trypanosomiasis you inject trypanosomiasis medicine, after that you inject the medicine used for East Coast fever, if it is not responding I inject the medicine called Berelin*,^4^ *later on if the livestock is not responding it can die or with God's grace it can recover. –* Women's FGD participant, Village 17, agro-pastoral

**Q7**. *I: So when the livestock does not get better you go and get another medicine and inject? Rs: Yes*.

*I: So you try different medicine until [you get] the one that responds? Rs: Yes. R8: When the livestock gets better you don't know which medicine worked*.

*I: That's a problem, how do you know the medicine that cured it, it might be the medicine that you used earlier [that] cured it? So, you just go on guessing? Rs: Yes*.

*I: So you don't have any adviser?*

*R1: There is no expert that is close*. –Women's FGD participants, Village 13, agro-pastoral

As the quotes demonstrate self-treatment decisions and options are often contingent on past experience and shared knowledge, past experimentations, and not uncommonly, a lack of other options.

#### Self-Treatment Based on Advice

While perceptions of self as expert were common among respondents, self-treatment does not preclude seeking advice either from formal health providers or from their own social networks.

***Q8***. *R1: When I go to this elder I explain the signs on my cattle and he can tell me I treated my livestock on same disease this way and using certain cc (cubic centimetre) so I go to do the same. I: So you take knowledge from a person who has experienced the same problem? R3: We use that way. –*Women's FGD participants, village 17, agro-pastoral

In some cases, participants reported travelling to an agrovet to buy medication and while there, they may ask for advice on the course of treatment:

**Q9**. *I: What if it's a new disease you have never seen, are you still going to treat yourself?*

*R1: That is where the problem is*.

*R4: You just treat saying maybe its tryps or CBPP/CCPP [Contagious Bovine Pleuropneumonia/Contagious Caprine Pleuropneumonia]*

*R7: It means you get to the shop and explain yourself. The LFO will tell you to take this medicine then inject maybe a certain cc according to the instruction, so when you get home you inject according to the instruction given*. – Women's FGD participants, Village 13, agro-pastoral

People recognise that formal experts sometimes also are guessing, as they are not basing advice on direct diagnostics or even clinical evaluation but reported conditions from livestock keepers. Participants also reported being able to treat livestock themselves due to having received advice prior from a formal health provider (such as an LFO or agrovet):

**Q10**. *R: By the time you go to buy medicine you will have already talked to the doctor [LFO] at a certain point and treated your livestock so you will have learned something from that. So, when the livestock gets sick suddenly and you don't have the doctor's communication you take action by following the instructions that you got earlier*. –Women's FGD participants, Village 20, agro-pastoral

So, while participants reported being able to administer medication themselves, in many cases they still rely on expert advice to do so.

#### Preference for Local Practise

Building off past experience, prior advice, trial and error, and cultural norms and practises, livestock keepers build up a skillset that demonstrates clear preferences for some types of treatments over others. Quite often, people directly expressed their preference for lay treatments, for using herbal remedies, or self-diagnosis and treatment. Use of herbs, roots, barks, other local remedies, and traditional management practises (herein all referred to as “local remedies”) are commonplace across field sites, however there was a greater propensity for local remedies in pastoralist communities, where there is often pride in knowing how to treat both human and livestock ailments rooted in traditional or local environmental knowledge.

**Q11**. *I: Are there other traditional medicines that are used to treat livestock when they get sick?*

*R1: For me I remember only those I have mentioned for treating Olodokulak [babesiosis]*.

*R2: For livestock who have retained the placenta they were given a drug called Olemudong'o […] yes you go to the forest/bush, take those medicines then you boil it and leave it to cool then you give to an animal with that problem. I2: Are you all using the same traditional medicines? Rs: Yes*

*R3: [For] a cow with Nunuk [swollen feet or lethargy] we usually apply ashes on it is back*. – Womens' FGD participants, Village 6, pastoral

#### Contingencies and Conditionalities of Self-Treatment

While the findings suggest that there may be an apparent preference to self-treat this preference is conditional on a suite of factors mediated by existing sociocultural knowledge *and* extrinsic structural constraints. The propensity for livestock keepers to treat livestock themselves is influenced by factors such as access (e.g., prohibitive costs of formal treatment, lack of service availability) and trust in the health care system (e.g., historic relationships to the state, trust in competence) and through preference for local knowledge/remedies.

#### Lack of Acces

Self-treatment *and* notions of expertise are driven in part because people do not have ready access to formal treatment options, such as LFOs or trained veterinarians serving their herds. This lack of access is either because of the costs and financial constraints or lack of expertise located in a convenient (or even remotely geographically accessible) location.

**Q12**. *R: I prefer to treat myself, since the government does not come to provide service, I do not have a person to rely on*. – FO participant 57, pastoral

This contradiction is particularly apparent in pastoralist communities, where local knowledge and sense of self-expertise is strong and where political and economic marginalisation is also extensive.

#### Costs

Costs are also a prohibitive factor when deciding whether to use formal health providers. Often, LFOs are only able to visit a sick livestock if they deem the service to be financially viable in order to offset expenses incurred via fuel, medicines etc:

**Q13**. *I: Do you have a livestock officer here (in the village)?*

*Rs: There is no livestock officer*.

*I: How about in the ward?*

*Rs: S/he [sic] is present. R4: To call for him is costly*.

*I: So the main reason people don't use the livestock officer is the cost? Rs:Yes. R4: And you can call him/her but until s/he comes the livestock will have died because he does not come the same day, he stays two to three days without coming so you can't wait for him. R3: If you call him and inform him about two or one livestock he might not come. R4: He wants the number of livestock to be big*.

*I: So he wants the number to be big for him to come?*

*R3: Yes*.

*I: But if it is two cattle?*

*R3: If it is two cattle he won't come. I:Even if you pay him?*

*R1: Maybe if paid he will come, under your cost*. –Women's FGD participants, Village 17, agro-pastoral

**Q14**. *R: Because now it is like a business, so when you phone a livestock doctor s/he will respond immediately*.

*I: Really?*

*R: Because s/he will earn money, they are doing business and they are not helping for free*. –Men's FGD participants, Village 23, pastoral

Thus, the combination of costs, the uneven ratios of service provision and livestock across the region, and varying levels of infrastructure for transportation or communication highlight that there are multiple ways that health inequities can occur.

#### Trust in Treatments, Providers, and the Health System

In some cases, a sense of self as expert casts doubt on the treatment capabilities of formal providers, compared to the experience of village elders. This belief reinforces the sentiment that they themselves are livestock experts and therefore are best placed to decide on appropriate treatments for their livestock, but also highlights the dangers of being too reliant on others who might prove to be unreliable:

**Q15**. *R: Even I can treat myself if the doctor tells me that the medicine is this I can treat alone. You know there is a medicine which is not allowed to be administered intramuscularly or others [that] are administered subcutaneously*.

*The vet should just instruct me how to treat*.

*I: Why?*

*R: You know there is a Swahili proverb:*

“*Mtegemea cha nduguye hufa masikini” which means that “He who relies on his brother's property dies poor,” so it is good if you know yourself*. – FO participant 25, pastoral

What at first glance then seems to be a preference for local knowledge and a confidence in self-expertise, soon reveals a much more complex narrative:

**Q16***.I: You treat it yourself? R2: We don't have a livestock officer here we treat ourselves. R5: We treat our self. I: So you are all doctors? R2: We are doctors. I: Or the seller gives you instruction? R3: If you ask, he will give you instruction. I: There is no time when you call the livestock officer? R4: None. R2: You call the livestock officer if the problem has become big, meaning in your boma* [compound where extended household and livestock reside*] many livestock are sick but if it is one or two you treat. R6: The problem is that the doctor can be called but he cannot treat better than this elder. I: This elder can have more experience than the livestock officer? R6: He knows more than the livestock officer that is in this area [...] maybe the district people know that a certain disease has erupted and [they] use the ward or district livestock officer, or there is vaccination to be done that is when he does the work. But in the boma [homestead] of this elder he goes to the medicine shop to explain his cattle's sickness and he is given medicine. R5: Or he goes and asks for a certain disease using his experience, so we are not at that point of waiting until the livestock officer tells you it is a certain disease*. –Men's FGD participants, Village 9, pastoral

The “preference” for self-treatment is also rooted in long standing relationships with the state established at independence. Different state regimes were associated with either showing livestock keepers *how* to treat livestock (e.g., introduction of clinical veterinary services during the Nyerere era) or for the dependence on self-reliance due to withdrawal of state services in rural areas (a result of changing governance to public services) as presented in the quote below:

**Q17**. *R1: Yes, we just inject the teremice*^5^ *[sic] (with luck) and God will heal an animal*.

*I: From where or whom have you learned how to administer the drug to an animal by injection? Or you have observed the LFO or livestock doctor doing that? Rs: Laugh. R1: Who taught us before? R2: Nyerere! It is Nyerere who was the first to use this way of treating animals with these artificial drugs. [Other respondents laugh]. R3: What? Nyerere was the one taught us how to administer the drug via injection? R2: No but he [his government] was the first to bring livestock medicines*.

*I: Okay and how about injection, the specific area to be injected by that medicine or drug you were shown by Nyerere?*

*R2: I know it myself. R1: Sometimes we learn from other people who know how and where to inject animal then later you will go to inject your animals*.

*I: So you learn from other people?*

*Rs: Yes. R2: You know I am not lying when I said Nyerere because the cattle have the first injection in Nyerere regime*.

*I: Yes. R2: After Nyerere injected the cattle in the [cattle] crush every one of us observed and from there we learned to how conduct an injection to our animals. -*

*-*Women's FGD participants, Village 6, pastoral

While President Nyerere is associated with introducing biomedicine equitably through socialised care,^6^ subsequent state regimes have left livestock keepers feeling resigned to the lack of services, and thus the impetus to keep treating livestock themselves due to lack of alternatives.

**Q18**. *I: Are you happy with the livestock services that are available in your area?*

*R: Is just that we are already used to it but we are not happy because other livestock die a lot without knowing what is killing them but because we don't have an alternative we are happy [to do] what can we do. –* FO participant 3, agropastoral

The complicated history of certain social groups to the state has, at times, also manifested in mistrust in formal experts and in biomedical products (particularly vaccination) as seen in the quotes below:

**Q19***.R1: He doesn't want [to teach us to do our own vaccinations], and if he does teach us, he can give you fake medicine. R2: He does not bring fake medicine. R1: Honestly, he brings fake (or expired) medicine, there are cattle of ours which he vaccinated and many of them died, those which didn't die, the area [on their body] that was injected had insects [sic] coming out. –*Men's FGD participants, Village 18, agro-pastoral

**Q20**. *R: We also think those people who manufacture medicine are business oriented. When you give the cattle certain medicine [there] has to come a time [when the] medicine [becomes] outdated and you have to go and buy again. We suspect even when you give livestock deworming medicine that is when the worms reproduce more. At that time when you give them the medicine the worms will die but when the worms become full again you have to go back to buy the medicine. –*Women's FGD participant, Village 20, agro-pastoral

In some cases, previous negative experiences with biomedicine, such as problematic vaccination campaigns in which animals died and during which incomplete information was provided about risks, can lead to participants believing formal providers lack the appropriate skills and technical capacity to administer medication.

**Q21**. *R: The government should bring good experts for testing cattle because, for example, the person who vaccinated cattle [which] then got humps doesn't see that it has caused the citizens not to have faith in the government. Like today, many people did not bring cattle [to the sampling site] because of the vaccination done [in the past] and it is just a person who made a mistake. The government should plan well when bringing those people for vaccination and they should give us experts that vaccinate cattle at a level that is required*. –Women's FGD participant, Village 20, agro-pastoral

Thus, self-reliance has limitations, is conditional, and when self-treatment options are exhausted, people move on to formal treatments, if they can.

***Q22***. *I: So people don't use her [LFO] because she's far or they already know how to treat so they don't see the reason of using her?*

*R1: No, it's not because she's far, if you have a problem at your house you go to her, and she has a vet shop, so when you find her at the shop you explain [your problem] to her, buy the medicine and she gives instructions. I: So she is the one at the vet shop?*

*Rs: Yes, she's the one that sells*.

*I: So when you go to the shop she is one who gives all the instruction? Rs: Yes*.

*R5: When you fail completely she does the follow up*.

*I: That is when she comes here? Rs: Yes*.

*I: So people use the LFO when they have failed to treat [themselves]? Rs: Yes. R4: When she gives you medicine and it does not work she changes it, when you fail she comes to your home to check the livestock and treat them. R7: When you call this LFO to come and check the cattle for diseases, it is expensive, you have to pay, and that's why many people are afraid to use her. R1: We can't use her, it's very expensive, if you don't have money what are you going to do?* –Women's FGD participants, Village 13, agro-pastoral

### Formal Treatment as Diversified HSB Experiences: The Narratives

As self-treatment often involves a succession of treatments with livestock keepers gauging the effectiveness of the option at each step, formal treatment is most commonly engaged at the “end” or as a last resort within a livestock keeper's HSB process. When options are exhausted, and no positive changes in an animal's conditions are observed, an LFO (where available) is called to come and examine or diagnose the sick animal. In addition to the last resort problem, seeking formal expertise also occurs when conditions arise that participants do not feel they have sufficient capabilities to manage on their own e.g., for unfamiliar diseases, wider disease outbreaks, or known acute illnesses such as anthrax. This however is not always the situation across all study sites. Smallholder (including agro-pastoral and peri-urban communities) or where livestock keepers more commonly have exotic breeds and fewer livestock, LFOs are called more regularly as first line treatment and the sense of “self as expert” is not as pronounced as in other areas, and many perceive the stakes as being too great to *not* call a vet. However, in our study communities where the rates of endemic zoonoses are highest, i.e., pastoralist then agro-pastoralist communities (2, 5) formal treatment via LFOs is still the last step in the HSB process and the sense of “self as expert” was expressed more often amongst pastoralist respondents [see also Mangesho et al. (43)]. Thus, like self-treatment, formal treatment is often conditional and influenced by cost, severity of the health condition, personal sense of ability to treat, and availability of services; and formal treatment is most commonly used as a last resort measure.

#### When All Other Options Are Exhausted and the Agrovet Options Don't Work, i.e., the Last Resort

While agrovets are often the first line of formal treatment (i.e., for purchasing drugs) they are also used as sources of advice about drugs or conditions livestock may have. This process tends to be provisional on (a) prior advice from another expert or past experience; (b) availability and convenience; and (c) costs. Agrovets are also commonly only sought after some initial consultation (with elders, with others in one's household) or after failing to achieve improvement or resolution using medicines already at home. These scenarios are evidenced in the quotes below:

**Q23***.I: How many times do you treat until you change the medicine? R: When you put the medicine and see that the livestock is not changing you can go and look for instruction from the livestock doctor and say there is a livestock of mine that is in this [or that] condition, I have given this medicine and it's (condition) is not changing, what other medicine should I try? You will hear him … try this, that is the explanation of the livestock officer. –*FO participant 15, agro-pastoral

**Q24***.I: So what you do is that when you see a livestock is sick you go to the shop, explain to the seller and he gives you directions for treatment? R3: Even by using this phone I can call the livestock officer, explain the condition of the livestock which then he explains to me what to do, I take the medicine and use the measurements explained to me. I: Is it costly to call* [phone] *the livestock officer? Rs: Yes. –*Mixed FGD participants, Village 22, agro-pastoral

**Q25***.R3: You see the livestock officer. I: So livestock officer has to come and see the livestock? R6: You explain to him the situation. I: You phone him? R6: You can phone him and explain the situation and he will advise you on which medicine to use or you can go to the livestock medicine shop and explain the condition of your livestock then they can provide service. –*Men's FGD participants, Village 10, agro-pastoral

The LFO coming to one's house for evaluation and treatment is the ultimate last resort, and only occurs if trust between the community and veterinary services or government exists.

**Q26**. *R: You have to go through that process since you are looking for any way for treatment, so if you get angry [that there are no services] you will ruin or lose your livestock*.

*I: Ok, if it happens that some other time your livestock get a [serious] problem … will you call the vet or?*

*R: When you look at it, I can't do it with my own knowledge, at times a different condition might happen and I see that this medicine that I am using can't treat that disease and I have to go and see the doctor/vet or livestock officer, to do the follow up. [This happened and…] I explained to him the diseases that they had so he came with his medicine and gave it to the livestock, and after that it [the illness] did not continue and the other livestock recovered*. –FO participant 15, agro-pastoral

When this trust has broken down, LFOs may not be used, even as a last resort.

***Q27***. *R: When the doctor is administering the medicine he must be sure the medicine will help, also that the customer and the livestock keeper are satisfied. Sometimes someone might say the doctor treated [your animal] so why are the livestock still not in good condition? So if he is not sure of what he's doing that's when there will be a competition (between doctor and livestock keeper), and maybe the doctor does not see the importance of the livestock like I do*. –Men's FGD participants, Village 20, agro-pastoral

#### Regular LFO Use

In the few cases where participants stated they rely primarily on LFOs, even for general malaise, it is in villages where they are readily available or are supplemented by community livestock health workers and other non-state paraprofessionals. Thus, the lines of “formal” treatment are again blurry as recognition of paraprofessionals varies district-to-district.

Vaccinations and specific disease outbreaks also serve as mediating forces in HSB for professional, formal care, including care from district officers or sometimes researchers. In these instances, people always rely on the LFOs, though this is in part because diagnosis or vaccines are not available/for sale to livestock keepers directly, as seen in Q14.

Finally, the use of self-treatment options and their surrounding contingencies does not necessarily preclude a desire for more access to expertise and professional assessment, either in the form of an actual service provider or more information or education that can facilitate further, more effective self-treatment. However, this too is predicated on past experiences and trust with the system.

**Q28**. *I: So for example when a livestock is sick would you like to call a doctor to treat or you will treat yourself*.

*R: If the doctor is near I would like to call him to come and inspect and test the livestock*.

*I: Why do you like to call the doctor and not treat yourself?*

*R: He's an expert*. –FO participant 20, agro-pastoral

Throughout the interviews and FGDs people expressed a desire for more services, better care, and more interaction (including seminars and education) from district/government veterinarians and extension officers.

## Discussion

Craddock and Hinchliffe (54) point to the need for in-depth qualitative methods to be an integral part of One Health research because of the unique ability of social sciences to foreground uneven geographies; frame health problems in terms of suffering and loss and not just risk; analyse relationships and situations that produce precarious bodies in the first place; and foreground the voices of those experiencing health and illness. The adoption of more in-depth, nuanced approaches that situates individual health behaviours within the contexts in which people live, as advocated in this study, has allowed us to describe the complex, non-linear and contingent narratives of HSBs. This process has allowed us to reveal the multiple, interconnected bio-social factors (such as agency, access, and trust) which influence health-related decision-making which discrete categories within KAP studies may fail to show, as they tend to focus on awareness of specific diseases, risks, or conditions.

The ways people “think and act” cannot and should not be distilled down to individual “pieces of information” [(55), p. 154]. Situating individual decision-making, actions, or “knowledge” within the contexts in which health and illness occur can reveal people's abilities or sense of agency to be able to care for their livestock, and which are part of broader bio-social systems. These issues are not always acknowledged by biomedical audiences. Thus, the idea commonly promoted through health interventions (and KAP studies as well) is often that one's sense of agency will change merely with good advice or the right information, i.e., through increased “knowledge” and thus health interventions can be directed towards these gaps in knowledge (56, 57), disease risks can be mitigated [as for zoonoses, see Zhang et al. (58) for review], or at a minimum stimulate discussion for improving health outcomes (59). However, we recognise agency and ability to shift behaviour is mediated not just through knowledge acquisition or shifting attitudes, but through social interactions and broader political-economic landscapes. We therefore suggest that in-depth social science can help reveal heterogeneity of local practise, values, and socially constructed realities that mediate health choices while also shaping human-non human relationships, especially where the porous boundaries between humans and non-humans can affect disease risks and health experiences. Because we “share our social, political, and medical landscapes with numerous biological beings,” governance of zoonoses, for example, cannot be concerned with human health alone [(60). p. 6]. These aspects of health are too easily neglected in health policies and within health systems. It is not only One Health approaches that point to the importance of recognising these linkages, but so do more holistic approaches to health and well-being (8, 61).

From our findings, we suggest that health seeking behaviours are constructed from a limited set of options that people face with limited capabilities and within which *access* and *trust* arise as paramount factors in the process. Such factors are not necessarily captured within KAP-style studies yet are critical to influencing how people can act when responding to livestock illness. *Access* to and *trust* in health care options/systems in turn affect patients and livestock keepers' individual and collective agency to affect change and positive health outcomes for themselves and their livestock.

### Access

**Figure 3:**
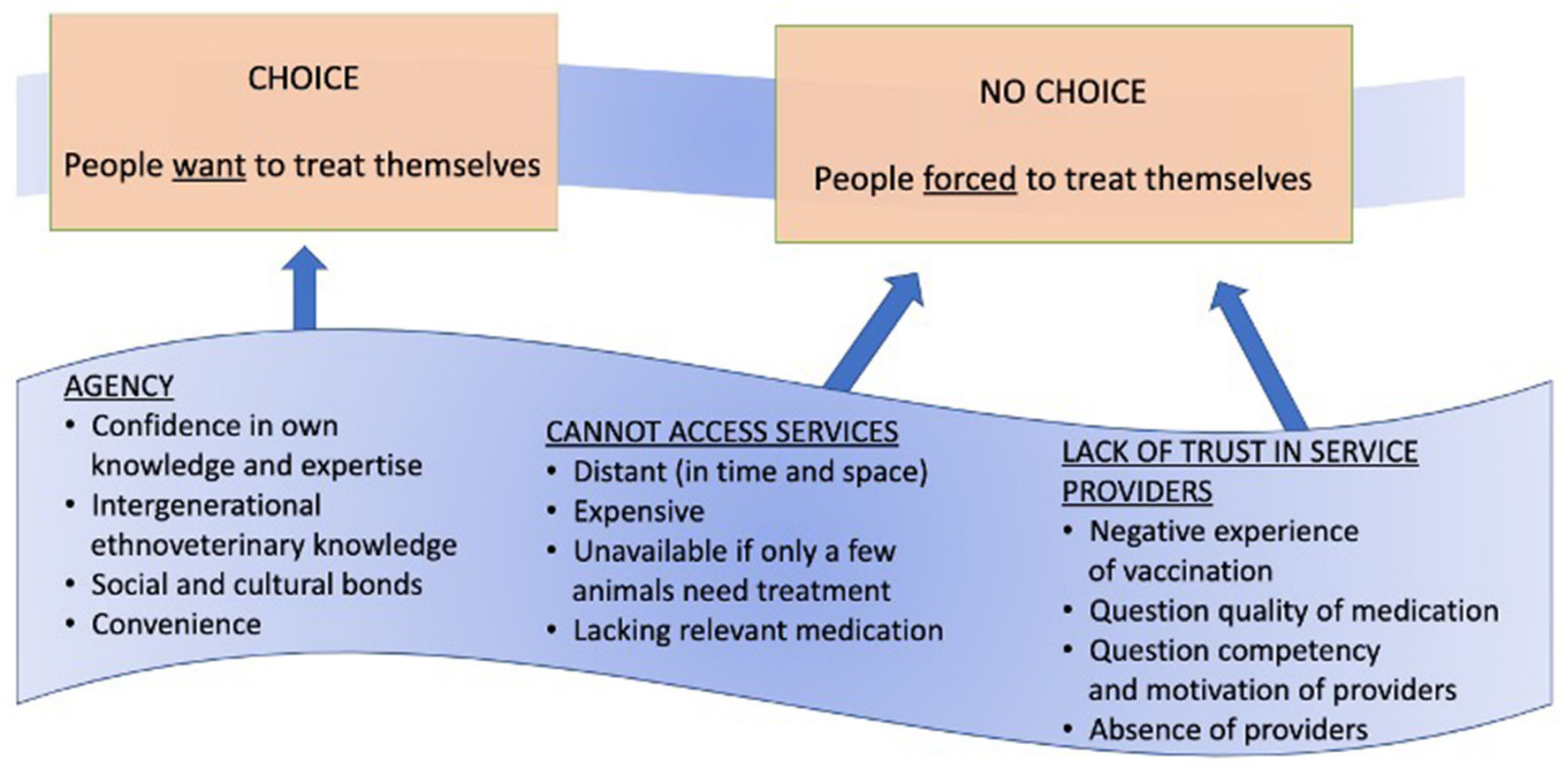
Agency, access and trust as interdependent aspects of health seeking behaviours.

Our data demonstrates communities define and access expertise and care in complex and contingent ways. Therefore, the ability for people to make “good” or rational decisions within the constraints they face (their agency) is not always straightforward or singular (see Figure 3). For example, while some respondents choose to self-treat due to perceptions of “self as expert” (and therefore feel they have no need to access formal services), in some cases this is a “false preference” and is directly linked to poor provision of health services within one's community. While many livestock keepers have deep generational knowledge, observational skills, and the cultural knowledge and experiences as livestock experts (43, 62), this does not lessen the significant sentiment (that many participants expressed) that the reason they self-treat is due to lack of available professionals in the area or prohibitive costs when they are available. While some LFOs are reachable via phone to offer advice, they are rarely able to visit the sick livestock or administer treatment due to limited infrastructure, transport, value for money and high work demands. In general, they are described by participants as being distant (in time and space) or completely absent, expensive, unavailable, or inaccessible. When they are available, they are often seen to be lacking in appropriate diagnostic supplies or drugs. However, this is not universal and communities of smaller geographic size, closer proximity to cities/towns, and of specific livelihoods (i.e., smallholders) tend to have better access to services. This is true for both human and livestock health services. Thus, access is impacted by broader health structure inequalities that are found at micro and macro scales within and outside Tanzania. Moreover, people's definitions and experiences often contrast with official policies and structures, such as the public-private provisions in Tanzania's 2006 Livestock Policy (34). In most communities, accessing care is a complex process and contingent on multiple factors.

### Trust

Our data also reveals how HSB and service access are linked to issues of trust in the care experience overall. As such, increased availability of LFOs or government services would not necessarily result in increased utilisation. Participants' trust in and perceptions about available health services plays a key role in influencing the decision to use them. Trust is tied to a community's past experience with government services, and sometimes is linked to long histories with the state that span past colonial and postcolonial administrations. Trust is also linked to an individual or community's belief in the service provider's competence (e.g., treatment capabilities, knowledge and skills), their motivations (e.g., being business oriented rather than genuine desire to help), or in the quality of drugs administered. Additionally, the responsiveness (or lack of responsiveness) when formal service providers are called add to people's levels of trust in the system. Historical relationships to the state, to formal services (health and beyond) also shape people's acceptance, use, and reliance on it (8). When LFOs or providers are expedient, available and helpful (in providing care, information, or education), this builds trust, reliance and use. When these are absent, this leaves participants little choice other than to rely on themselves to administer treatment to their livestock themselves, or to not treat livestock at all. Thus, the self-reliance and self perception of expertise and “choice” that people have built into their HSB are not only about access to but also trust in the services that are available.

### Agency

Recognising the importance of access and trust within health seeking behaviour highlights the limits of people's agency in their health seeking behaviours. We stress that it is critical to recognise that people's sense of agency is multi-dimensional. For example, on the one hand, agency can be a component of empowerment, where people experience a sense of self sufficiency, confidence, and belief in their own knowledge, experience and expertise and as such are able to make decisions that improve livestock health outcomes. Yet this can butt against the limitations of access to services, like diagnostics or vaccines that require formal, professional expertise. On the other hand, negative experiences with the livestock health system and a sense of lack of choice or poor choices can hamper decision options or health outcomes. Thus, while we saw various aspects of agency play out across all field sites, as stated above, an overreliance on personal choice, empowerment and livestock keeper agency in livestock health outcomes should not draw attention away from the key structural inequalities of health that persist in human and veterinary health systems in Tanzania or globally.

### Structural Inequities

Recognising that the existence of possible courses of actions is dependent upon wider issues of prior experience and trust in formal systems of provision highlights the need to take into account those factors that lead to individuals developing a sense of active agency. Our findings and analysis suggest that linking health choices to broader factors that shape these choices (and hence binding issues of agency to trust and access) can help further frame HSB and health interventions in East Africa and beyond. Likewise, structural inequalities of health are not just bound to national and global inequities but can also be tied to structural issues within particular health systems themselves, or within communities, households and families which have their own hierarchies of power, cultural norms, and practises that affect day-to-day health care decisions and options (63, 64). Rylko-Bauer and Farmer (65) take care to link not only structural inequalities to structural violence (a now long emphasised view of the seriousness, pervasiveness, and embeddedness of health in broader structural factors), but also to suffering, which further humanises the concept. These authors also link Sen's notions of agency (66) to structural violence of health stating it is vital to see the ways agency is constrained by the “matrix of culture, history, and political economy” [(65), p. 52] and how this is in turn linked to suffering. To address structural violence of health, suffering, likewise needs to be seen, and yet, it often remains silent or “invisible” [(65), p. 52, (67)] though it is in plain view, it is just not “dramatic.” This may be doubly so for the unseen suffering of animals (outside the well-recognised effects of livestock health on people and their livelihoods). The lack of health services, the acceptance of “self-expertise” in lieu of other options, are normalised and undramatic, and may seem “empowering” when they may be the opposite. How can health services be better addressed, more evenly distributed to communities who need it, and yet who may be resistant to increased attention due to histories of poor trust? We argue for seeing and addressing both the structural inequity of health at global, national, and local scales as well as for more provider engagement with the communities they serve in order to improve health services provision and access and which will contribute to improved trust and empowerment, particularly for veterinary based HSBs.

### Empowerment

Empowerment, as a development of sense of agency to enact change, is bound to complex and intertwined factors such as *access and trust* as they are in other facets of life (8, 68). Individual and community empowerment is bound to accessing one's rights as well as one's history, past experiences (either positive or negative), knowledge, and belief that one can make effective change or have a positive impact (69). However, empowerment alone is insufficient to improve health outcomes or meet health needs (for either people or their livestock). While the ability to make “good” health decisions may be a critical component of health justice (69) it still places the central control of health outcomes into *individual* decision making. Our approach demonstrates the need to look beyond individual behaviours and to scrutinise more thoroughly the contextual and structural factors that influence the extent to which an individual is able to act. As we have highlighted in this discussion, issues relating to access and trust become critical threads throughout these decisions (70).

## Conclusion

Our research demonstrates how structural inequalities of health may be reproduced through health seeking behaviours, misplaced notions of individuality, agency, and empowerment in HSB and the reproduction of structural factors that inhibit an individual's ability to act. We paid particular attention to health choices and options for livestock keepers and their livestock and demonstrated the need to be mindful of broader and immediate contextual factors that impact health and well-being. We point to how access and trust are key factors in HSB, and how these tie into issues of structural inequalities of health. Finally, we argue for more engaged, in-depth social science research of (veterinary) health to move beyond individual KAP based studies, draw out the complex factors that shape behaviour, and bring attentiveness to the role of the wider social contexts within which human and animal health occur.

## Data Availability Statement

The original contributions presented in the study are included in the article/supplementary material, further inquiries can be directed to the corresponding author/s.

## Ethics Statement

The studies involving human participants were reviewed and approved by Kilimanjaro Christian Medical Centre (KCMC/832) and National Institute of Medical Research (NIMR/2028) in Tanzania, College of Medical, Veterinary and Life Sciences at the University of Glasgow (39a/15). Approval for study activities for each researcher was also provided by the Tanzanian Commission for Science and Technology (COSTECH) and by the Tanzanian Ministry of Livestock and Fisheries, as well as by regional, district, ward and village-level authorities in the study area. The patients/participants provided their written informed consent to participate in this study.

## Author Contributions

AD, JV, JS, SC, BM, JC, ES, WG, and JH contributed to the conceptualisation and design of the study. JS, SC, JB, and JC contributed to funding acquisition. AD, FL, KT, ES, WG, KM, TM, and MT were responsible for data curation, investigation, analysis, and project administration. AD, JV, and JS performed analysis and wrote the first draught of the manuscript. ES, WG, TM, SC, JH, KT, JC, and FL contributed writing and revision of the manuscript. All authors have approved the submitted version.

## Funding

This study was supported by the Biotechnology and Biological Sciences Research Council, Department for International Development, the Economic and Social Research Council, the Medical Research Council, the Natural Environment Research Council and the Defence Science and Technology Laboratory funded this research under the Zoonoses and Emerging Livestock Systems (ZELS) programme (BB/L018926/1 and BB/L018845/1). The funders had no involvement in the study design, data collection, analysis, or interpretation of the findings. The funders played no role in writing or submitting this paper.

## Conflict of Interest

The authors declare that the research was conducted in the absence of any commercial or financial relationships that could be construed as a potential conflict of interest.

## Publisher's Note

All claims expressed in this article are solely those of the authors and do not necessarily represent those of their affiliated organizations, or those of the publisher, the editors and the reviewers. Any product that may be evaluated in this article, or claim that may be made by its manufacturer, is not guaranteed or endorsed by the publisher.

## Footnotes

^1^We specifically refer to “health inequity” rather than “health inequality.” The former denotes an unjust and unfair distribution of health risks and resources, whereas the latter refers to any measurable aspect of health that varies across individuals or social groups. Health inequality is absent from moral judgement on whether the differences are fair or just (31).

^2^Wards are administrative units, smaller than districts and larger than villages. Wards are often comprised of 3–5 villages, which are based on human population.

^3^Respondents (plural) are referred to as “Rs”, individual respondents as “R” and Interviewer as “I” throughout. If there are multiple interview respondents they will be numbered, i.e., R1, R2.

^4^Berenil® - Diminazene Aceturate and Phenazone granules for an injectable solution against trypanosomiasis in cattle. Commonly referred to as “Berelin” in Maasai/Swahili usage.

^5^Terramycin® is a brand name for an injectable oxytetracycline however it has been shortened to “teremice” in the Swahili/Maasai usage.

^6^Participants often compared the socialist pre-SAP state under president Nyerere to current state regimes, the former associated with nostalgic times of post-independence nation building and unity, and the latter which are associated with worsened economic conditions and diminished public and veterinary health infrastructure.

## References

[1] Pica-Ciamarra U Baker D Morgan N Ly C Nouala S. Business and Livelihoods in the Livestock Sector in Africa: Investments to Overcome Information Gaps. World Bank (2014). Available online at: https://cgspace.cgiar.org/handle/10568/35161 (accessed June 10, 2021).

[2] de Glanville WA Davis A Allan KJ Buza J Claxton JR Crump JA. Classification and characterisation of livestock production systems in northern Tanzania. PLoS ONE. (2020) 15:e0229478. 10.1101/2020.02.10.941617 33378382 PMC7773236

[3] Covarrubias K Nsiima L Zezza A. Livestock and Livelihoods in Rural Tanzania : A Descriptive Analysis of the 2009 National Panel Survey. Washington, DC: World Bank. ©World Bank. (2012). Available online at: https://openknowledge.worldbank.org/handle/10986/17886 (accessed May 10, 2021). License: 1158 CC BY 3.0 IGO.

[4] Cleaveland S Sharp J Abela-Ridder B Allan KJ Buza J Crump JA. One health contributions towards more effective and equitable approaches to health in low- and middle-income countries. Philos Trans R Soc B Biol Sci. (2017) 372:20160168. 10.1098/rstb.2016.0168 28584176 PMC5468693

[5] Halliday JEB Allan KJ Ekwem D Cleaveland S Kazwala RR Crump JA. Endemic zoonoses in the tropics: a public health problem hiding in plain sight. Vet Rec. (2015) 176:220–225. 10.1136/vr.h798 25722334 PMC4350138

[6] Ahmed H Yoder J de Glanville WA Davis A Kibona TJ Mmbaga BT. Economic burden of livestock disease and drought in Northern Tanzania. J Dev Agric Econ. (2019) 11:140–51. 10.5897/JDAE2018.1028

[7] MacGregor H Waldman L. Views from many worlds: unsettling categories in interdisciplinary research on endemic zoonotic diseases. Philos Trans R Soc B Biol Sci. (2017) 372:20160170. 10.1098/rstb.2016.0170 28584178 PMC5468695

[8] Davis A Sharp J. Rethinking One Health: emergent human, animal and environmental assemblages. Soc Sci Med. (2020) 258:113093. 10.1016/j.socscimed.2020.113093 32531688 PMC7369629

[9] Caudell MA Dorado-Garcia A Eckford S Creese C Byarugaba DK Afakye K. Towards a bottom-up understanding of antimicrobial use and resistance on the farm: a knowledge, attitudes, and practices survey across livestock systems in five African countries. PLoS ONE. (2020) 15:e0220274. 10.1371/journal.pone.0220274 31978098 PMC6980545

[10] Ndagala DK. Pastoralists and the State in Tanzania. Nomadic Peoples. (1990) 25–27:51–64.

[11] McCabe JT Leslie P Davis A. The emergence of the village and the transformation of traditional institutions: a case study from Northern Tanzania. Hum Organ. (2020) 79:150–60. 10.17730/1938-3525.79.2.150 33551464 PMC7861511

[12] Fratkin E. East African pastoralism in transition: Maasai, Boran, and Rendille Cases. Afr Stud Rev. (2001) 44:1–25. 10.2307/525591

[13] Hinchliffe S Bingham N Allen J Carter S. Pathological Lives: Disease, Space and Biopolitics. 1st ed. Chichester, West Sussex; Malden, MA: Wiley-Blackwell (2016).

[14] Bardosh K. One Health: Science, politics and zoonotic disease in Africa. 1st ed. Oxon: Routledge (2016).

[15] Konadu-Agyemang K. IMF and World Bank Sponsored Structural Adjustment Programs in Africa: Ghana's Experience, 1983-1999. London: Routledge (2018).

[16] Farmer P Basilico M Kleinman A Kim JY. Reimagining Global Health: An Introduction. 1st ed. Berkeley: University of California Press (2013).

[17] Obrist B Iteba N Lengeler C Makemba A Mshana C Nathan R. Access to health care in contexts of livelihood insecurity: a framework for analysis and action. PLoS Med. (2007) 4:e308. 10.1371/journal.pmed.0040308 17958467 PMC2039761

[18] Montgomery CM Mwangee W Kong'ong'o M Pool R. ‘To help them is to educate them': power and pedagogy in the prevention and treatment of malaria in Tanzania. Trop Med Int Health. (2006) 11:1661–9. 10.1111/j.1365-3156.2006.01719.x 17054745

[19] Muela SH Ribera JM Toomer E Grietens KP. The PASS-model: a model for guiding health-seeking behavior and access to care research. Malaria Rep. (2012) 2:3. 10.4081/malaria.2012.e3

[20] Awosanya EJ Akande HO. Animal health care seeking behavior of pets or livestock owners and knowledge and awareness on zoonoses in a university community. Vet World. (2015) 8:841–7. 10.14202/vetworld.2015.841-847 27047163 PMC4774675

[21] G/hiwot TT Sime AG Deresa B Tafese W Hajito KW Gemeda DH. Community health seeking behavior for suspected human and animal rabies cases, gomma district, Southwest Ethiopia. PLoS ONE. (2016) 11:e0149363. 10.1371/journal.pone.0149363 26959816 PMC4784896

[22] MacKian S. A Review of Health Seeking Behaviour: Problems and Prospects. Health Systems Development Programme, University of Manchester (2003). Available online at: https://assets.publishing.service.gov.uk/media/57a08d1de5274a27b200163d/0503_health_seeking_behaviour.pdf (accessed May 19, 2021).

[23] Hausmann-Muela S Ribera J Nyamongo I. No. 14 Health-Seeking Behaviour and the Health System Response. (2003). Available online at: https://www.semanticscholar.org/paper/No-.-14-Health-seeking-behaviour-and-the-health-Hausmann-Muela-Ribera/35116a3c0a88898a531ce214e4448861ae00e320 (accessed April 11, 2021).

[24] Launiala A. How much can a KAP survey tell us about people's knowledge, attitudes and practices? Some observations from medical anthropology research on malaria in pregnancy in Malawi. Anthropol Matters. (2009) 11:1–13. 10.22582/am.v11i1.31

[25] Farmer P. Infections and Inequalities: The Modern Plagues. Berkeley: University of California Press (2001)

[26] Mangesho PE Caudell MA Mwakapeje ER Ole-Neselle M Kimani T Dorado-García A. Knowing is not enough: a mixed-methods study of antimicrobial resistance knowledge, attitudes, and practises among maasai pastoralists. Front Vet Sci. (2021) 8:645851. 10.3389/fvets.2021.645851 33834048 PMC8023390

[27] Bardosh K. Global aspirations, local realities: the role of social science research in controlling neglected tropical diseases. Infect Dis Poverty. (2014) 3:35. 10.1186/2049-9957-3-35 25320672 PMC4197218

[28] Thornton PK Herrero MT Freeman HA Okeyo Mwai A Rege JEO Jones PG. Vulnerability, climate change and livestock - opportunities and challenges for the poor. J Semiarid Trop Agric Res. (2007) 4:1–23. Available online at: http://www.icrisat.org/journal/SpecialProject/sp7.pdf

[29] Farmer P. Pathologies of power: rethinking health and human rights. Am J Public Health. (1999) 89:1486–96. 10.2105/AJPH.89.10.1486 10511828 PMC1508789

[30] Parker M Polman K Allen T. Neglected tropical diseases in biosocial perspective. J Biosoc Sci. (2016) 48 (Suppl. 1):S1–S15. 10.1017/S0021932016000274 27428062

[31] Arcaya MC Arcaya AL Subramanian SV. Inequalities in health: definitions, concepts, and theories. Glob Health Action. (2015) 8:27106. 10.3402/gha.v8.27106 26112142 PMC4481045

[32] Mujinja PGM Kida TM. Implications of Health Sector Reforms in Tanzania: Policies, Indicators and Accessibility to Health Services. Dar es Salaam: Economic and Social Research Foundation (2014). Available online at: https://www.thdr.or.tz/docs/THDR-BP-8.pdf (accessed March 29, 2021).

[33] Kitandu LZ. Animal health governance and services: a case of pastoralists in Ngorongoro district, Tanzania. ABC Res Alert. (2014) 2. 10.18034/abcra.v2i3.289

[34] United Republic of Tanzania. National Livestock Policy. The Ministry of Livestock and Development (2006). Available online at: https://www.tnrf.org/files/E-INFO_National_Livetock_Policy_Final_as_per_Cabinet_Dec-2006.pdf

[35] Mlangwa JED Kimera IE Magayane FT. Veterinary paraprofessionals and community animal health workers in Tanzania. Tanzan Vet J. (2008) 25:121–31. 10.4314/tvj.v25i2.42035

[36] Auty H Swai E Virhia J Davis A de Glanville W Kibona T. How can we realise the full potential of animal health systems for delivering development and health outcomes? Rev Sci Tech. (2021) 40:483–495. 10.20506/rst.40.2.3239 34542101

[37] Kariuki RW Munishi LK Courtney-Mustaphi CJ Capitani C Shoemaker A Lane PJ. Integrating stakeholders' perspectives and spatial modelling to develop scenarios of future land use and land cover change in northern Tanzania. PLoS ONE. (2021) 16:e0245516. 10.1371/journal.pone.0245516 33577608 PMC7880460

[38] Goldman MJ Riosmena F. Adaptive capacity in Tanzanian Maasailand: changing strategies to cope with drought in fragmented landscapes. Glob Environ Change. (2013) 23:588–597. 10.1016/j.gloenvcha.2013.02.010 25400331 PMC4230704

[39] Galvin KA. Transitions: pastoralists living with change. Annu Rev Anthropol. (2009) 38:185–98. 10.1146/annurev-anthro-091908-164442

[40] BurnSilver SB Worden J Boone RB. Processes of Fragmentation in the amboseli ecosystem, Southern Kajiado District, Kenya. In: Galvin K. A. Reid R. S. R. H. B. Jr Hobbs N. T., editors. Fragmentation in Semi-Arid Arid Landscapes: Consequences for Human Natural Systems. Dordrecht: Springer Netherlands. p. 225–253.

[41] Homewood K. Ecology of African Pastoralist Societies. Oxford : Athens, OH : Pretoria: Ohio University Press (2008).

[42] Queenan K Mangesho P Ole-Neselle M Karimuribo E Rweyemamu M Kock R. Using local language syndromic terminology in participatory epidemiology: lessons for One Health practitioners among the Maasai of Ngorongoro, Tanzania. Prev Vet Med. (2017) 139:42–9. 10.1016/j.prevetmed.2017.02.003 28364831

[43] Mangesho PE Caudell MA Mwakapeje ER Ole-Neselle M Kabali E Obonyo M. “We are doctors”: drivers of animal health practices among Maasai pastoralists and implications for antimicrobial use and antimicrobial resistance. Prev Vet Med. (2021) 188:105266. 10.1016/j.prevetmed.2021.105266 33517159

[44] Langwick SA. Bodies, Politics, and African Healing: The Matter of Maladies in Tanzania. Illustrated edition. Bloomington: Indiana University Press (2011).

[45] Virhia J. Healthy animals, healthy people: lived experiences of zoonotic febrile illness in northern Tanzania. (PhD thesis). University of Glasgow, UK (2019). Available online at: http://theses.gla.ac.uk/79058/ (accessed June 1, 2021).

[46] URT. The Veterinary Act. Dar es Salaam: Government of Tanzania (2003).

[47] OIE. OIE Competency Guidelines for Veterinary Paraprofessionals. Paris: OIE World Organisation for Animal Health (2018).

[48] Mokgobi MG. Understanding traditional African healing. Afr J Phys Health Educ Recreat Dance. (2014) 20:24–34. 26594664 PMC4651463

[49] Robinson TP Thornton PK Franceschini G Kruska RL Chiozza F Notenbaert. Global Livestock Production Systems. Rome: Food and Agriculture Organization of the United Nations (FAO) and International Livestock Research Institute (ILRI) (2011). 152 p.

[50] United Republic of Tanzania. National Bureau of Statistics. Population Distribution by Administrative Areas. (2012). Available online at: https://www.nbs.go.tz/index.php/en/censussurveys/population-and-housing-census/162-2012-phcpopulation-distribution-byadministrative-areas (accessed May 30, 2021).

[51] United Republic of Tanzania. National Bureau of Statistics. National Sample Census of Agriculture (2002/2003). Available online at: https://books.google.co.uk/books/about/National_Sample_Census_of_Agriculture_20.html?id=3UeSjgEACAAJ&redir_esc=y

[52] Braun V Clarke V. Using thematic analysis in psychology. Qual Res Psychol. (2006) 3:77–101. 10.1191/1478088706qp063oa

[53] Bernard HR. Handbook of Methods in Cultural Anthropology. 2nd ed. Lanham: Rowman & Littlefield Publishers (2014).

[54] Craddock S Hinchliffe S. One world, one health? Social science engagements with the one health agenda. One World One Health Soc Sci Engagem One Med Agenda. (2015) 129:1–4. 10.1016/j.socscimed.2014.11.016 25434985

[55] Pelto PJ Pelto GH. Studying knowledge, culture, and behavior in applied medical anthropology. Med Anthropol Q. (1997) 11:147–63. 10.1525/maq.1997.11.2.147 9186958

[56] Tillyard G DeGennaro V. New methodologies for global health research: improving the knowledge, attitude, and practice survey model through participatory research in Haiti. Qual Health Res. (2019) 29:1277–86. 10.1177/1049732318816675 30565510

[57] Holt HR Eltholth MM Hegazy YM El-Tras WF Tayel AA Guitian J. Brucella spp. infection in large ruminants in an endemic area of Egypt: cross-sectional study investigating seroprevalence, risk factors and livestock owner's knowledge, attitudes and practices (KAPs). BMC Public Health. (2011) 11:341. 10.1186/1471-2458-11-341 21595871 PMC3121632

[58] Zhang N Zhou H Huang D-S Guan P. Brucellosis awareness and knowledge in communities worldwide: a systematic review and meta-analysis of 79 observational studies. PLoS Negl Trop Dis. (2019) 13:e0007366. 10.1371/journal.pntd.0007366 31048848 PMC6497230

[59] Nicoll A Laukamm-Josten U Mwizarubi B Mayala C Mkuye M Nyembela G. Lay health beliefs concerning HIV and AIDS–a barrier for control programmes. AIDS Care. (1993) 5:231–241. 10.1080/09540129308258604 8329487

[60] Wolf M. Is there really such a thing as “one health”? Thinking about a more than human world from the perspective of cultural anthropology. One World One Health Soc Sci Engagem One Med Agenda. (2015) 129:5–11. 10.1016/j.socscimed.2014.06.018 24961737 PMC7131074

[61] Zinsstag J Schelling E Waltner-Toews D Tanner M. From “one medicine” to “one health” and systemic approaches to health and well-being. Prev Vet Med. (2011) 101:148–56. 10.1016/j.prevetmed.2010.07.003 20832879 PMC3145159

[62] Caudell MA Quinlan MB Subbiah M Call DR Roulette CJ Roulette JW. Antimicrobial use and veterinary care among agro-pastoralists in Northern Tanzania. PLoS ONE. (2017) 12:e0170328. 10.1371/journal.pone.0170328 28125722 PMC5268417

[63] Bourgois P Holmes SM Sue K Quesada J. Structural vulnerability: operationalizing the concept to address health disparities in clinical care. Acad Med J Assoc Am Med Coll. (2017) 92:299–307. 10.1097/ACM.0000000000001294 27415443 PMC5233668

[64] Chandler CIR. Current accounts of antimicrobial resistance: stabilisation, individualisation and antibiotics as infrastructure. Palgrave Commun. (2019) 5:1–13. 10.1057/s41599-019-0263-4 31157116 PMC6542671

[65] Rylko-Bauer B Farmer P. Structural violence, poverty, and social suffering. Oxf Handb Soc Sci Poverty. (2016) 47–75. 10.1093/oxfordhb/9780199914050.013.4

[66] Sen A. Rights and agency. Philos Public Aff. (1982) 11:3–39.

[67] Scheper-Hughes N. Small wars and invisible genocides. Soc Sci Med 1982. (1996) 43:889–900. 10.1016/0277-9536(96)00152-9 8870153

[68] Davis A Goldman MJ. Beyond payments for ecosystem services: considerations of trust, livelihoods and tenure security in community-based conservation projects. Oryx. (2019) 53:491–6. 10.1017/S0030605317000898

[69] Goldman MJ Davis A Little J. Controlling land they call their own: access and women's empowerment in Northern Tanzania. J Peasant Stud. (2016) 43:777–97. 10.1080/03066150.2015.1130701

[70] Mohseni M Lindstrom M. Social capital, trust in the health-care system and self-rated health: the role of access to health care in a population-based study. Soc Sci Med. (2007) 64:1373–83. 10.1016/j.socscimed.2006.11.023 17202025

